# The Donation of Human Biological Material for Brain Organoid Research: The Problems of Consciousness and Consent

**DOI:** 10.1007/s11948-024-00471-7

**Published:** 2024-02-05

**Authors:** Masanori Kataoka, Christopher Gyngell, Julian Savulescu, Tsutomu Sawai

**Affiliations:** 1 Graduate School of Humanities and Social Sciences, Hiroshima University, Higashi-Hiroshima, Japan; 2 Biomedical Ethics Research Group, Murdoch Children’s Research Institute, Melbourne, Australia; 3 Melbourne Law School, The University of Melbourne, Melbourne, Australia; 4 Department of Paediatrics, The University of Melbourne, Melbourne, Australia; 5 Centre for Biomedical Ethics, Yong Loo Lin School of Medicine, National University of Singapore, Queenstown, Singapore; 6 Faculty of Philosophy, University of Oxford, Oxford, UK; 7 Institute for the Advanced Study of Human Biology (ASHBi), Kyoto University, Kyoto, Japan

**Keywords:** Brain organoids, Consciousness, Consent, Autonomy, Precautionary principle

## Abstract

Human brain organoids are three-dimensional masses of tissues derived from human stem cells that partially recapitulate the characteristics of the human brain. They have promising applications in many fields, from basic research to applied medicine. However, ethical concerns have been raised regarding the use of human brain organoids. These concerns primarily relate to the possibility that brain organoids may become conscious in the future. This possibility is associated with uncertainties about whether and in what sense brain organoids could have consciousness and what the moral significance of that would be. These uncertainties raise further concerns regarding consent from stem cell donors who may not be sufficiently informed to provide valid consent to the use of their donated cells in human brain organoid research. Furthermore, the possibility of harm to the brain organoids raises question about the scope of the donor’s autonomy in consenting to research involving these entities. Donor consent does not establish the reasonableness of the risk and harms to the organoids, which ethical oversight must ensure by establishing some measures to mitigate them. To address these concerns, we provide three proposals for the consent procedure for human brain organoid research. First, it is vital to obtain project-specific consent rather than broad consent. Second, donors should be assured that appropriate measures will be taken to protect human brain organoids during research. Lastly, these assurances should be fulfilled through the implementation of precautionary measures. These proposals aim to enhance the ethical framework surrounding human brain organoid research.

## Introduction

Organoids are three-dimensional tissues derived from stem cells, such as embryonic stem cells or induced pluripotent stem cells, that are used to replicate the process of organogenesis *in vitro*. Among these, “brain organoids” specifically mimic the brain. The first brain-like tissues resembling the cerebrum were created from human embryonic stem cells in 2008 (Eiraku et al., 2008) and were later termed “organoids” in 2013 (Kadoshima et al., 2013; Lancaster et al., 2013). Since then, researchers have produced organoids that mimic various brain regions, including the cerebrum, hypothalamus, and pituitary gland. However, current brain organoid technology faces several limitations. Notably, a lack of blood vessels restricts the size of brain organoids to that of a pea or smaller. Additionally, they differ significantly from the normal brain in terms of maturity, laminar structure, absence of an input/output system, and lack of supporting cells (Benito-Kwiecinski & Lancaster, 2020).

Despite these limitations, brain organoids are valuable tools for studying the developmental processes of the brain and modeling diseases. To date, brain organoids have played an important role in studying microcephaly caused by Zika virus infection during pregnancy (Dang et al., 2016). Furthermore, brain organoids may have future applications in regenerative medicine. It may be possible to grow region-specific brain organoids to replicate damaged parts of a human brain and subsequently transplant them to restore proper function. Human brain organoids have already been successfully transplanted into the brains of mice, rats, and rhesus macaques (Chen et al., 2019; Kitahara et al., 2020; Revah et al., 2022).

Various ethical issues have been identified in brain organoid research (Sawai et al., 2022). Many of these issues relate to the possibility that brain organoids possess some form of consciousness. The concept of “consciousness” has various meanings and has been a subject of in-depth and vigorous debate within the fields of philosophy and cognitive science (Michel, 2020). However, nearly all scientists and ethicists believe that, at present and in the near future, brain organoids do not possess any form of consciousness owing to their structural and functional simplicity (International Society for Stem Cell Research, 2021). Moreover, it remains uncertain whether conscious brain organoids can ever be created. Thus, discussions regarding the possible consciousness of brain organoids are largely speculative. Nevertheless, it is important to consider the ethical implications of this possibility to anticipate future problematic research scenarios and enhance public trust in scientific research.^1^

If brain organoids were to exhibit consciousness, how should brain organoid research be approached? We refer to this as “the problem of consciousness.” While this problem holds inherent importance, in this paper, we specifically examine how it complicates the issue of obtaining consent from human cell donors for brain organoid research. In Sect. “The Problem of Consciousness: Double Uncertainty”, we provide a more detailed introduction to the problem of consciousness. Subsequently, in Sect. “The Complicated Problem of Consent”, we provide an overview of previous discussions that have linked the problem of consciousness to consent. They have discussed how the problem of consciousness creates challenges in respecting the donor’s autonomy during the consent procedure. In Sect. “Moral Uncertainty and the Scope of Autonomy”, we highlight further how it may transform brain organoid research into a research category that could harm third parties (i.e., organoids). This kind of harm falls outside the scope of donor autonomy or self-determination. Therefore, adequate ethical oversight is necessary to prevent donors from inadvertently participating in unethical research, and the details of ethical oversight need to be communicated to the donors to protect their moral integrity. Considering these complications, in Sect. “Proposals for the Consent Procedure”, we briefly present three proposals regarding the consent procedure for brain organoid research. First, project-specific informed consent should be implemented. Second, donors should be informed that appropriate measures will be taken to mitigate the risks associated with brain organoids. Third, precautionary measures should be implemented to ensure the well-being of donors and minimize potential harm.

## The Problem of Consciousness: Double Uncertainty

The potential consciousness of brain organoids poses a significant ethical concern in brain organoid research. If brain organoids were to achieve consciousness like that of human or animal, they would possess moral status, necessitating careful consideration of the ethical implications when subjecting them to invasive manipulations and eventual destruction. Determining whether and in what sense brain organoids can be conscious is a crucial task, but it is complicated by two uncertainties, epistemological and moral uncertainties (Sharma et al., 2021).

### Epistemological Uncertainty

The first uncertainty is epistemological. Currently, there is limited scientific knowledge about the presence and forms of consciousness in future brain organoids. There is no established scientific method for determining whether and how a neural network realizes consciousness. The ongoing controversy among various theories of consciousness makes it unlikely that a consensus will be reached soon. As a result, determining the consciousness of brain organoids is highly dependent on the theory of consciousness one endorses (Lavazza, 2020; Niikawa et al., 2022; Zilio & Lavazza, 2023). For example, according to the global workspace theory, consciousness can arise when there is functional connectivity between cortices through long-range projections (Dehaene & Naccache, 2001). Current brain organoids do not meet this condition, and it will not easily be met in the future. Alternatively, the integrated information theory posits that the intrinsic ability of a system to integrate information is a sufficient condition for consciousness (Tononi et al., 2016). According to this theory, even current brain organoids with certain complex patterns of neural activity could be considered conscious. Many other rival theories of consciousness make different claims about the consciousness of brain organoids (Niikawa et al., 2022).

Most theories of consciousness would agree that current immature and simple brain organoids are not conscious. Therefore, epistemological uncertainty is relatively low at present. However, if more complex brain organoids can be cultured in the future, uncertainty will increase. Epistemological uncertainty regarding brain organoids may be a long-term feature of decision-making in this field.^2^

### Moral Uncertainty

Even if the state of consciousness in brain organoids could be determined, a second uncertainty arises: the relationship between the different forms of consciousness and the moral consideration is unclear. It is important to distinguish the meanings of the term “consciousness.” An entity may be conscious in the sense of having experiences that are neither good nor bad for that entity,^3^ in the sense of having good or bad experiences for that entity like pleasure or pain (being sentient), in the sense of having advanced cognitive abilities, and so forth.^4^ However, the moral significance of these features and to what degree they matter remain uncertain.

Sentience, which refers to the capacity for good or bad experiences, and other more advanced forms of consciousness are relatively ethically unambiguous. This is because we are already familiar with examples of sentient non-human animals and cognitively advanced humans, and there is some agreement on how we should treat these entities in research. Thus, we can say that brain organoids should be treated *at least* in the same way as animals or humans if they have animal- or human-like sentience or advanced cognitive abilities.^5^

However, there is no consensus on the moral significance of other forms of consciousness, such as experiences that are neither good nor bad for their possessor. Furthermore, as brain organoid technology advances in the future, the moral importance of such experiences will become a pressing question. Researchers have divergent views on this issue. Some argue that only good or bad experiences are morally worth considering (Shepherd, 2018), while others contend that experiences without goodness or badness also hold moral significance (Niikawa, 2018). As the discussion on the moral significance of this form of consciousness has only recently begun, it is unlikely that a consensus will be reached soon.

Although many would agree that it is *pro tanto* wrong to invade or discard an entity with some form of consciousness^6^ it is uncertain whether the treatment of brain organoids falls under such wrongdoing. Therefore, brain organoid research is characterized by moral uncertainty. This moral uncertainty is currently less relevant, as many theories of consciousness would agree that current brain organoids lack consciousness. However, this uncertainty will also become salient as brain organoid technology develops.

Thus, the problem of consciousness presents a double uncertainty that complicates ethical discussions on the topic. In this paper, we do not attempt to solve this problem. Instead, we focus on how this uncertainty complicates the issue of consent from cell donors.

## The Complicated Problem of Consent

The problem of consciousness in brain organoid research is often regarded as a novel problem in bioethics, while consent for brain organoid research is regarded as a classical ethical problem (Lavazza & Massimini, 2018; Koplin & Savulescu, 2019). Although consent is a traditional issue in bioethics (Faden & Beauchamp, 1986), new problems often intersect old problems. The problem of consciousness may present challenges to obtaining consent for brain organoid research. The challenges can be categorized into two types: challenges for broad consent and challenges for project-specific consent.^7^ While the focus differs, the core issue remains the same: the uncertainties inherent in brain organoid research make it difficult to respect the donor’s autonomy or right to self-determination during the consent procedure (Sugarman & Bredenoord, 2019; Hyun et al., 2020; Greely, 2021).

### Challenge for Broad Consent

The problem of consciousness may undermine the validity of broad consent in biobanks and similar institutions. The human biological materials provided to biobanks may be used for various studies over a long period. Informing donors of the details of future research at the time of initial consent is extremely difficult, if not impossible. For this reason, some biobanks adopt the method of broad consent, whereby consent is given for an unspecified range of future uses under certain restrictions (Grady et al., 2015). Therefore, broad consent allows for the use of samples in future diverse medical research endeavors. However, it has been argued that this should not extend to brain organoid research (Boers & Bredenoord, 2018; Farahany et al., 2018). For example, Greely (2020) argues that broad consent is insufficient for “controversial uses”, including brain organoid research, which some donors may object to. Greely suggests that donors should be asked to provide explicit consent based on specific details of the research project for such uses. If cells for which broad consent has already been obtained are to be used, consent should be obtained retrospectively (Greely, 2020).^8^ Greely does not explicitly state why brain organoid research would be controversial, but we can assume that the problem of consciousness is a plausible source of controversy. Research that may produce novel conscious entities is distinct from standard medical research, and it is not surprising if some donors object to the possibility of conscious entities being created from their cells.^9^  

A comprehensive understanding of donors’ attitudes toward brain organoids requires empirical investigation. To date, there is only one relevant study (Haselager et al., 2020). This study conducted semi-structured interviews with 28 Dutch citizens and found that many interviewees were willing to donate their cells for brain organoid research if sufficient information was provided. However, when instructed to consider the possibility of conscious brain organoids, moral concerns emerged. While many individuals were opposed to brain organoids with advanced cognitive abilities, others were opposed to those with *any* form of consciousness owing to the following concerns: the devaluation of human uniqueness, misuses, and the welfare of the conscious brain organoids. Moreover, those who attributed greater moral significance to brain organoids called for stricter ethical constraints on the research involving their cells. Thus, this study suggests that, for some donors, broad consent does not imply consent for brain organoid research.^10^ However, it is important to note that this is only a tentative claim based on a small-scale study. Further empirical investigations into donors’ attitudes toward brain organoids are required to deepen the discussion.

### Challenge for Project-Specific Consent

For project-specific consent, the problem of consciousness in brain organoid research poses another challenge. Owing to the aforementioned double uncertainty, researchers would face difficulties in providing donors with definitive explanations regarding the details of brain organoid research. This may result in inadequate informed consent, raising doubts about its validity (Greely, 2021; Mollaki, 2021). Certainly, informing individuals about uncertainties is an important component of informed consent, and accepting a high degree of uncertainty is part of the donor’s right to self-determination (Deuring, 2022). Donors need to be informed of empirical information about organoids’ capacities and nature relevant to their own values around moral status. Then, some donors may be able to resolve the moral uncertainties based on their personal moral standards.^11^ However, when the degree of uncertainty becomes very high, it may compromise the validity of the consent.

As mentioned previously, there are currently relatively little epistemological and moral uncertainties regarding immature brain organoids. If this situation persists, valid consent to organoid research is possible. However, if more complex brain organoids can be cultured in the future, the emergence of morally significant consciousness becomes more uncertain. Consequently, obtaining valid informed consent will become increasingly challenging.

## Moral Uncertainty and the Scope of Autonomy

Previous discussions linking the problem of consciousness to consent have highlighted how uncertainty makes it difficult for donors to make autonomous decisions. However, even if certain types of consent can respect the donor’s autonomy, another problem arises. The moral uncertainty of brain organoids may place the research partially outside the proper scope of the donor’s autonomous decision-making.

In research that poses various risks to the participants, obtaining their consent is necessary. These risks may include physical harm, disclosure of sensitive information, deception, and challenges to one’s beliefs. In such cases, it is reasonable to consider that if the participants are fully informed and consent to the research, the risks involved are largely (if not entirely) justified. This is what it means to respect the participants’ autonomy or right to self-determination.

In contrast, the risks of brain organoid research may not only involve the donors but also extend to third parties, i.e. the brain organoids. Brain organoid research has the potential to create entities with morally significant consciousness and subsequently harm or destroy those entities. This is precisely the sense in which the research is morally uncertain. As the risks to these third parties are not risks to the participants, they fall beyond the appropriate scope of the participants’ autonomy or self-determination.

It is not uncommon for individuals to consent to participate in research involving risks to third parties. However, in such cases, the research cannot be fully justified by consent alone. A relevant example is human-animal chimera research, which involves not only human cell donors but also laboratory animals. For such research, ethical oversight regarding animal welfare, in addition to the consent of the participants, is critical. The necessity of using animals must be rigorously evaluated, and even when animal experimentation is deemed necessary, efforts should be made to minimize animal suffering. The consent from donors does not establish the reasonableness of the risks and harms involved in the research, which ethical oversight must establish. This role of ethical oversight is especially important if the cell donors themselves have little interest in the informed risks or harms in animal welfare. We believe the same principles should apply to future brain organoid research.^12^ A donor who is informed about the details of a particular brain organoid study may willingly accept the high degree of moral uncertainty based on their own moral standards. However, this does not reduce the moral uncertainty of the research itself. Thus, respecting donors’ autonomy is not the sole problem in the consent procedure for brain organoid research. What is overlooked is the problem of *consenting to unethical research*, which makes the donor complicit.

Without adequate consideration for the possible welfare of the brain organoids, donors may have a moral reason to refrain from donating their cells. Again, drawing an analogy with human-animal chimera research is helpful. Individuals may have firm moral views related to the moral status of chimeric animals, that ought normally to be respected, and information relevant to these must be provided. However, suppose a cell donor is informed that the creation and use of chimeric animals will cause harm to them, but there is no consideration for the animals’ welfare. The donors may still agree to donate cells to this study based on their own moral standards, but they should not do so in this case. There are moral reasons for the donor not to donate their cells, leading to unjustified harm to the animals. To prevent donors from becoming complicit in unethical research in such a way, researchers should inform donors that the welfare of the animals will be appropriately considered and implement measures to ensure their welfare.

We believe that the same should apply to cell donation for brain organoid research if future brain organoids become more mature and more likely to possess consciousness. Donors must be fully informed that measures will be taken to minimize potential harm to the organoids. This provides another reason for project-specific consent rather than broad consent.^13^ If individuals have not been informed of such measures, they should not donate their cells to such research, regardless of their beliefs regarding the moral importance of brain organoid consciousness. Notably, as mentioned previously, there is a double uncertainty regarding whether and how research may harm brain organoids. These uncertainties create ambiguity regarding the specific actions that would be implemented to mitigate possible negative consequences. Nevertheless, this does not imply that nothing can be done (see Proposal 3 in Sect. “Proposals for the Consent Procedure”).

In summary, we argue that the donor’s consent is morally unproblematic and sufficiently justifies the use of donated cells in brain organoid research only if the organoids are treated appropriately. This supports the general concerns raised by Boers et al. (2016), who have pointed out that an emphasis on consent alone may not provide sufficient moral justification for using human tissues in organoid technology. They highlight the inherent problems associated with the storage, distribution, and use of organoids, primarily in the context of medical applications, and considerations of benefits for patients and personal information. We further emphasize that, in the case of the brain organoid technology, it is crucial to consider the possible harm to the organoids. Consent should not be used as an “ethics-wash” for deeply controversial research.

## Proposals for the Consent Procedure

The problem of consciousness presents significant challenges to obtaining informed consent in brain organoid research. To address these challenges, we present three tentative proposals for the consent procedure.

### Proposal 1: Develop Specific Consent Mechanisms

Broad consent is not sufficient for brain organoid research, as some donors may object to the possibility of creating a conscious entity from their cells. Additionally, it is crucial to inform the donor about the details of ethical oversight to ensure their active participation without unknowingly engaging in controversial research. Thus, to respect the donor’s autonomy, right to self-determination, and moral integrity, project-specific informed consent should be obtained (and re-obtained as much as possible) for each research project. In consent procedures, various aspects of uncertainty will need to be emphasized, as we explain in Proposal 2.

### Proposal 2: Incorporate Epistemological and Moral Uncertainties into Consent Procedures

When obtaining project-specific consent for brain organoid research, researchers should inform donors about the epistemological and moral uncertainties associated with the research. Donors should be given sufficient opportunity to critically reflect on these uncertainties. With current brain organoid research, we believe, it is acceptable for researchers to describe epistemological uncertainty (the uncertainty regarding the possibility that brain organoids can be conscious) as very low. However, if brain organoids become more complex, epistemological uncertainty would need to be continually revised. Furthermore, researchers should also stress the *moral* uncertainty involved (the uncertainty regarding the moral significance of various forms of consciousness). Donors should be fully informed that even if they accept higher levels of moral uncertainty, ethical oversight ensures that the research itself is (at least minimally) unproblematic. If these points are insufficiently stressed, there is a greater risk that the donated cells will be used contrarily to the donor’s autonomy and moral integrity.

### Proposal 3: Develop a Risk Framework for Brain Organoids

Safety and risk-mitigation measures must be implemented for brain organoids derived from donor cells, as explained to the donor in the consent procedure. In addition to the donor’s consent, these measures are necessary to morally justify the research.^14^ Ethicists and neuroscientists should collaborate to determine which types of research are more likely to result in conscious brain organoids that may experience suffering and find ways to mitigate such suffering. This should be incorporated into a risk framework to identify particularly risky forms of research.

Some authors have already recommended a precautionary approach to brain organoid research (Birch & Browning, 2021; Niikawa et al., 2022). According to these proposals, if there is a certain reasonable feasibility about the consciousness of brain organoids, research should be conducted *as if* they have morally significant consciousness. Specifically, research may be conducted with precautional adherence to principles such as the Three Rs (Replacement, Reduction, and Refinement) of animal experiments. These principles may inform the risk framework for brain organoid research.

However, further research is needed to address challenges in applying these principles to brain organoid research, such as how to compare the harms in animal research with those in brain organoid research. For example, it may be unclear how to reduce the suffering of brain organoids (Refinement). While it is relatively easy just to reduce the number of brain organoids to be created (Reduction), we must consider scenarios where such reduction leads to an increased reliance on animal research (Replacement). Handling situations where the only viable form of replacement amplifies the necessity for other morally precarious or problematic research poses a significant ethical challenge without clear guidelines.

Certain studies have suggested that brain organoid research should be subject to stricter restrictions beyond the Three Rs (Koplin & Savulescu, 2019). In a recent discussion on animal research, additional principles have been developed to protect animals more comprehensively (Beauchamp & DeGrazia, 2020). However, applying such new principles to brain organoid research is not straightforward. Apart from the challenges posed by the uncertainty of consciousness, each principle presents specific difficulties. For example, the Principle of Basic Needs requires meeting the basic needs of animals unless failure is unavoidable and morally justified. However, determining the basic needs of an artificially created entity such as a human brain organoid and how to fulfill them raise questions (Kataoka & Sawai, 2023).

Furthermore, the risks associated with brain organoid research may vary depending on which brain region is being recapitulated. Precautionary measures may need to be intensified for organoids that mimic brain regions known to be particularly relevant to consciousness, such as the cerebrum and brainstem.

It is also important to avoid oversimplifying the interpretation of precautionary measures. Assuming that brain organoids possess morally significant consciousness also carries risks (Żuradzki, 2021). For example, treating brain organoids with excessive ethical rigor could significantly stagnate research, with adverse long-term consequences for human health. This problem of competing risks is well recognized in the debate about the precautionary principle (Sunstein, 2005).

Therefore, while precautionary measures are crucial for brain organoid research, they may face numerous challenges. Prior to the culture of more mature brain organoids and the increased uncertainty regarding their consciousness, it is necessary to conduct detailed examinations on how to address these challenges. Such examinations should align with the discussion on the precautionary principle, animal research ethics, and the development of the brain organoid technology.

## Conclusions

The problem of consciousness in brain organoid research introduces significant uncertainty regarding the potential consciousness of brain organoids, the nature of that consciousness, and its moral implications. This uncertainty also extends to various other ethical issues. We have examined the impact of the problem of consciousness on the consent process and highlighted the challenges in respecting donor autonomy. We have also emphasized on the importance of ethical oversight and effective communication with donors regarding the potential consciousness of brain organoids. While autonomy is currently the primary concern given the immaturity of brain organoids and low uncertainty, as research progresses, ethical oversight will become increasingly important to prevent the misuse of consent in justifying controversial research. At that time, consent from donors should not be used to “ethics-wash” controversial research. Based on these considerations, we have presented three tentative proposals for an appropriate consent procedure. We have not provided a detailed examination of the desirable procedures but hope to stimulate further research and discussion. In future research, it is crucial to examine the limits of donor consent and develop a risk framework that guides organoid research, including the investigation of appropriate precautionary measures for brain organoids.

While we have focused on consent for brain organoid research, the arguments presented have broader implications for research involving (potentially) conscious entities, including animals. Informed consent requires comprehensive examination of the moral dimensions of the research proposed, ensuring that individuals do not unintentionally become involved in potentially unethical research. The ethical dimensions of research should always be considered in the consent procedure.

## Footnotes

1 Koplin (2023) argues that the potential improvement in cognitive abilities and moral status of human-animal chimeras (including animals implanted with human brain organoids) should be examined now, even if it may only be feasible in the future. The same could be said about the possibility that human brain organoids themselves could become conscious and, therefore, acquire an elevated moral status.

2 Here, we are not necessarily committed to the view that we *cannot* know about the consciousness of brain organoids or other entities in general. We are merely stating that it will be challenging in the near future. This difficulty may be resolved through developments in the science and philosophy of consciousness.

3 An entity may have visual, auditory, and other sensory experiences, but these experiences have no value to that entity. Brain organoids, which have areas related to vision but no areas related to rewards, may be an example of such an entity.

4 Experience (phenomenal consciousness), sentience, and advanced cognitive capacity are mentioned here because they have been featured in the literature on brain organoid ethics. However, they are just some of the various concepts used to capture different aspects of consciousness. For example, “wakefulness” and “awareness” are used to classify disorders of consciousness, but it is not always clear how these relate to the aforementioned concepts. While not the focus of this paper, further consideration of the moral significance of each diverse form of consciousness is necessary to reduce moral uncertainty. Such a detailed examination will also be important considering the possibility that brain organoids may only realize some of the conscious functions typically found in animals or humans. In such cases, it becomes challenging to simply compare the treatment of brain organoids with that of animals or humans, as we suggest below (thanks to a reviewer for this point).

5 Here, the caveat “at least” is important. Even if human brain organoids possess mouse-like consciousness, there may be reasons to believe that they deserve more moral consideration than mice. One possible reason is their human origin (Zilio & Lavazza, 2023). Although such a view can be seen as a form of speciesism — asserting a moral difference based solely on species distinction — we believe this claim cannot be easily dismissed in the context of the ethics of human biological materials. This paper presents several claims that analogize human brain organoids to animals, but these claims are minimal. We do not exclude the possibility that human brain organoids may hold greater moral importance than animals.

6 For simplicity, the phrase “*pro tanto*” is often omitted hereafter.

7 Additionally, it is suggested that considerations regarding consent procedures are insufficient for fully respecting the autonomy of donors in brain organoid research (Lewis & Holm, 2022).

8 For marketed human pluripotent stem cells, it would be unrealistic to obtain consent retrospectively from the donor for the creation of brain organoids. In such cases, ethical oversight becomes crucial to prevent the donor from unknowingly becoming complicit in unethical research (see note 13).

9 Brain organoid research may raise controversies beyond the issue of consciousness. For example, some authors suggest that informed consent will be important for the potential use of brain organoids in biocomputing (Kagan et al., 2023).

10 In a partially related study, 60 U.S. citizens were interviewed about their perspectives on organoids in general (Bollinger et al., 2021). Although this study did not specifically focus on brain organoids or cell donation, many interviewees perceived brain organoids as morally distinct from other types of organoids, partially owing to the possibility of consciousness.

11 For example, some individuals may not concern themselves with the moral status of brain organoids, much like those who do not consider the moral status of animals. However, even if donors can personally resolve the moral uncertainty this manner, it does not change the moral uncertainty of the research when assessed from the standpoint of research ethics (see the next section). Thanks to a reviewer for pointing this out.

12 If brain organoids possessed highly developed cognitive abilities that enabled them to make independent decisions, they would be considered research participants rather than entities like experimental animals. In such a scenario, the justification for conducting the research would necessitate obtaining consent from the cell donor and brain organoids. Sawai et al. (2022) highlighted that, even if the donor were to withdraw consent, the research may continue as long as it does not result in the destruction of the brain organoids (see also, Khuram et al., 2023). However, it is unlikely that brain organoids will reach such a sophisticated state in the foreseeable future.

13 As mentioned in note 8, it is difficult to re-obtain consent from the donor of marketed human pluripotent stem cells for the creation of brain organoids. This reinforces the importance of ethical oversight. Even if the donor consents to the use of their cells for any purpose, this does not justify involving them in unethical research.

14 This proposal is similar to the “consent for governance” model as it integrates research governance into the consent procedure. In the consent for governance model, the donor is informed about how future unspecified research will be governed, and the donated materials are used only for research that adheres to the agreed-upon governance conditions (Boers & Bredenoord, 2018). However, presently, when brain organoids are not widely understood by the society, it seems premature to adopt this model for brain organoid research. We believe that the possibility of creating conscious entities can be unexpected, even as something monstrous, to some donors. Even if the governance conditions are agreed upon, individuals without a thorough understanding of brain organoids may unwillingly become involved in the creation of potentially conscious entities, which can undermine their moral integrity. Therefore, we propose that, at least for the time being, project-specific consent should be individually obtained for brain organoid research.
